# Comparing the Yield of Nasopharyngeal Swabs, Nasal Aspirates, and Induced Sputum for Detection of *Bordetella pertussis* in Hospitalized Infants

**DOI:** 10.1093/cid/ciw521

**Published:** 2016-11-02

**Authors:** Marta C. Nunes, Nasiha Soofie, Sarah Downs, Naume Tebeila, Azwi Mudau, Linda de Gouveia, Shabir A. Madhi

**Affiliations:** 1 Department of Science and Technology/National Research Foundation, Vaccine Preventable Diseases; 2 Medical Research Council, Respiratory and Meningeal Pathogens Research Unit, University of the Witwatersrand; 3 National Institute for Communicable Diseases, National Health Laboratory Service, Centre for Respiratory Diseases and Meningitis, Johannesburg, South Africa

**Keywords:** *Bordetella pertussis*, nasopharyngeal swabs, nasopharyngeal aspirates, induced sputum infants, detection

## Abstract

***Background.*** Advances in molecular laboratory techniques are changing the landscape of *Bordetella pertussis* illness diagnosis. Polymerase chain reaction (PCR) assays have greatly improved the sensitivity detection and the turnaround time to diagnosis compared to culture. Moreover, different respiratory specimens, such as flocked nasopharyngeal swabs (NPSs), nasopharyngeal aspirates (NPAs), and induced sputum, have been used for *B. pertussis* detection, although there is limited head-to-head comparison to evaluating the PCR yield from the 3 sampling methods.

***Methods.*** Hospitalized infants <6 months of age who fulfilled a broad syndromic criteria of respiratory illness were tested for *B. pertussis* infection by PCR on paired NPSs and NPAs; or paired NPSs and induced sputum. An exploratory analysis of *B. pertussis* culture was performed on induced sputum specimens and in a subset of NPSs.

***Results.*** From November 2014 to May 2015, 484 infants with paired NPSs and NPAs were tested; 15 (3.1%) PCR-confirmed pertussis cases were identified, 13 of which were PCR positive on both samples, while 1 each were positive only on NPS or NPA. From March to October 2015, 320 infants had NPSs and induced sputum collected, and 11 (3.4%) pertussis cases were identified by PCR, including 8 (72.7%) positive on both samples, 1 (9.1%) only positive on NPS, and 2 (18.2%) only positive on induced sputum. The 3 types of specimens had similar negative predictive value >99% and sensitivity >83%. Compared to PCR, culture sensitivity was 60% in induced sputum and 40% in NPSs.

***Conclusions.*** Flocked nasopharyngeal swabs, nasopharyngeal aspirates, and induced sputum performed similarly for the detection of *B. pertussis* infection in young infants by PCR.

Infection by *Bordetella pertussis* affects all age groups, with young infants being most susceptible to developing severe disease [1, 2]. Timely and accurate diagnosis of pertussis illness could guide clinical management, including infection control in hospitals [3]. *Bordetella pertussis* isolation from a respiratory specimen by microbial culture is the conventional gold standard for confirming pertussis infection [4]. Culture, however, has a low sensitivity because the organism is fastidious. Furthermore, the sensitivity of culture decreases with illness progression, with the highest yield being during the catarrhal phase of illness, and it requires 7–10 days to grow, isolate, and identify the organism [5, 6]. Culture nevertheless provides an opportunity for phenotypic and genotypic characterization of *B. pertussis*. Polymerase chain reaction (PCR) is increasingly being used for the diagnosis of pertussis, as it provides rapid turnaround results and is more sensitive than culture during the latter part of illness and postantibiotic therapy [7, 8]. Careful selection of the target genes for PCR analyses is needed to achieve good specificity [9, 10].

Both the Centers for Disease Control and Prevention and the European Centre for Disease Prevention and Control recommend that specimens for testing of *B. pertussis* infection should be obtained by aspiration or swabbing the posterior nasopharynx [11, 12]. A number of studies have compared the yield from nasopharyngeal aspirates (NPAs) and flocked nasopharyngeal swabs (NPSs) for identifying respiratory viral or bacterial infections by PCR [13, 14]. Nasopharyngeal swab sensitivity of >90% compared to NPAs has been reported; posterior nasopharyngeal sampling is, however, a prerequisite for achieving detection rates comparable to those of NPAs [13, 15]. Induced sputum has also been explored in children as a specimen for respiratory pathogen detection in patients with lower respiratory tract infection (LRTI) and in adults with acute respiratory illness [16–19]. Two recent studies from South Africa found that in children <13 years old hospitalized for LRTI, induced sputum increased the diagnostic yield of *B. pertussis* over NPS alone by 50%–75% [19, 20].

In this study we compared the yield and sensitivity of NPS collected in universal transport media (UTM) with that of NPA and induced sputum for the detection of *B. pertussis* by PCR. In addition, we explored the sensitivity for *B. pertussis* culture when using NPAs or induced sputum.

## SUBJECTS AND METHODS

### Study Population and Sample Collection

**Figure 1.**
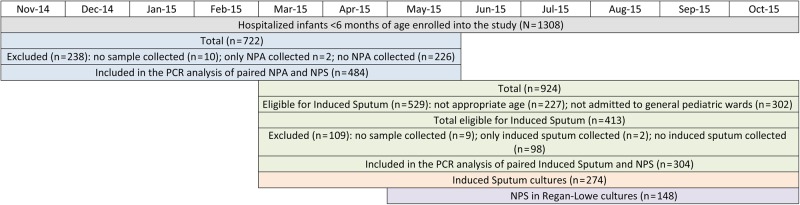
Diagram representing the number of infants enrolled and specimens analyzed during the study period. Abbreviations: NPA, nasopharyngeal aspirate; NPS, nasopharyngeal swab; PCR, polymerase chain reaction.

This study was nested in a hospital-based surveillance aimed at determining the pathogen-specific etiology associated with LRTI hospitalizations in children admitted to Chris Hani Baragwanath Academic Hospital (CHBAH) in Soweto, South Africa. This report describes the *B. pertussis* infections detected using different sampling methods in infants <6 months of age, enrolled from 10 November 2014 to 15 October 2015. The legal guardians for all hospitalized infants who fulfilled the broad syndromic criteria of respiratory illness—that is, any signs or symptoms of cough (irrespective of duration), tachypnea adjusted for age, lower chest wall recessions, cyanosis, apnea, or any other feature suggestive of a respiratory infection or neonatal sepsis—were approached for participation in the study. Detailed demographic and clinical information, including human immunodeficiency virus (HIV) exposure in utero, were collected from the hospital records and by interviewing the infants' primary caregivers. Enrolled infants had at least 1 NPS collected using a flocked swab for PCR testing. All infants included in the present analysis had an NPS collected and at least 1 other respiratory specimen (NPA or induced sputum). During the first period of the study, pertussis PCR–positive rates were compared between NPSs and NPAs; NPAs were collected from 10 November 2014 until 22 May 2015 (Figure 1). From 11 March 2015 to 15 October 2015, induced sputum was collected from infants aged 1 month to <6 months when clinically stable who were admitted to the general pediatric wards and excluding those admitted to the short-stay ward. Induced sputum specimens were tested by PCR and also plated on charcoal agar (Regan-Lowe agar, Media Mage, South Africa) for detection of *Bordetella* species by culture. An additional NPS was collected on Regan-Lowe transport media from participants <3 months of age from 25 May 2015 for detection of *Bordetella* species by culture. Figure 1 displays the number of the different sample types analyzed and the study dates.

Nasopharyngeal swabs were collected using a commercially available nylon flocked swab on the tip of a flexible plastic rod (FLOQS, Copan Flock Technologies, Brescia, Italy). The swab was gently passed up the nostril toward the pharynx for a distance equal to that between the patient's nares and earlobe, rotated 2–3 times, held in place for approximately 5 seconds, withdrawn gently, and placed in 2.5 mL UTM (FLOQS, Copan Flock Technologies); from May onward a second swab was collected from infants <3 months of age and placed in Regan-Lowe transport media. Nasopharyngeal aspirates were obtained by douching the nasopharynx using a 5-mL syringe with 2 mL of 0.9% sterile saline via a suction tube placed into 1 of the nostrils at the posterior aspect of the nasopharynx [21]. Mucus was suctioned out and collected in a sterile container with UTM. Induced sputum collection took place in a separate, well-ventilated room. For induced sputum collection sterility was maintained; infants were kept nil per mouth for 24 hours prior to the procedure and nebulized with a bronchodilator to open and prepare the airways. Chest massage was done to induce coughing up of sputum, and this was collected by suction into a sterile container. Specimens in UTM were transported on ice and the NPSs collected in Regan-Lowe at ambient temperature to the Respiratory and Meningeal Pathogens Research Unit laboratory (based at CHBAH), where bacterial culture was done immediately and PCR testing was performed within 24–48 hours.

### PCR Testing

Total nucleic acids were extracted from the clinical specimen using a NucliSENS easyMAG (bioMérieux, Marcy l'Etoile, France) platform and tested by qualitative real-time PCR for the presence of the multicopy pertussis insertion sequence (IS) *481* using a modified protocol to the one described by Tatti et al [9]; if IS*481* cycle threshold (Ct) values were ≤40, total nucleic acids were reextracted and tested by real-time PCR for IS*481* and in a duplex reaction for hIS*1001*and pIS*1001* with an annealing temperature of 57°C, and in a singleplex reaction for the pertussis toxin subunit S1 (*ptxS1*) with an annealing temperature of 60°C. The primers and probes (LTC Tech South Africa) used are listed in Supplementary Table 1. Internal controls were included to check the integrity of the samples and the efficiency of the extraction step and to detect the presence of PCR inhibitors. Positive controls were included in each experiment. We followed the 4-target algorithm developed by Tatti et al in our second PCR for determining PCR positivity for *B. pertussis* (Supplementary Table 2) [9]. Specimens with an intermediate PCR result were deemed as nonpositive in the current analysis.

### Culture

NPSs in Regan-Lowe media and induced sputum specimens were inoculated onto charcoal-enriched agar with 40 mg/mL cephalexin as well as 5% horse blood agar, upon arrival to the laboratory. Agar plates were incubated at 37°C in high-humidity ambient air, and observations on growth were made every 24 hours for up to 10 days. Catalase and oxidase reactions were performed on the suspected *Bordetella* colonies, and confirmatory PCR testing was done on the catalase- and oxidase-positive isolates. Colonies were identified based on typical morphology, growth on charcoal agar plates not present on corresponding blood agar plates, Gram stain, and catalase and oxidase testing. Those suspected to be *Bordetella* species were subjected to PCR testing.

### Statistical Analyses

The frequency of *B. pertussis* detection by PCR was compared between NPSs and NPAs and induced sputum specimens by χ^2^ test. Agreement between 2 sampling methods was assessed by Cohen κ, and values 0.8–1.0 were considered to have very good agreement. A composite reference standard was used to assess the sensitivity and negative predictive value (NPV) of each sampling method by considering any positive result from any of the specimens as the gold standard for presence of *B. pertussis*. This approach means that the specificity and positive predictive value of either sampling type by definition is 100%. Using this standard, we calculated sensitivity and NPV with 95% confidence intervals (CIs), and these were compared among specimens by Fisher exact test. Other continuous variables were represented as mean or median and compared by Student *t* test or Mann–Whitney test. Analyses were performed using Stata software version 13.1 (StataCorp, College Station, Texas) and GraphPad Prism version 6.00 (GraphPad, La Jolla, California). Statistical significance was concluded if *P* < .05.

### Ethics Consideration

The study was approved by the University of the Witwatersrand Human Research Ethics Committee (number 131109). The legal guardian(s) or parent(s) of the infants provided written informed consent for sample collection and clinical record review.

## RESULTS

Of the 1308 hospitalized infants <6 months of age enrolled into the study from 10 November 2014 to 15 October 2015, 711 (54.4%) were tested by PCR for *B. pertussis* by NPS and at least NPA and/or induced sputum; 50.9% (362/711) of these infants were aged <2 months. From November 2014 to May 2015, 484 infants with paired NPSs and NPAs were tested by PCR for *B. pertussis*; 238 infants enrolled during this period did not have paired NPSs/NPAs collected for testing (Figure 1). These 2 groups of infants were similar in sex distribution and age, although the HIV exposure status was unknown a for a higher percentage of infants without an NPA sample (2.1%) compared with infants with an NPA sample (0.4%) (*P* = .04; Table 1).

**Table 1.** Samples Available for *Bordetella pertussis* Polymerase Chain Reaction Testing During the Study Period

	November 2014 to May 2015		
Characteristic	NPA and NPS Tested by PCR (n = 484)	No NPA and NPS Tested by PCR (n = 238)	*P* Value
Age, d, at admission, median (IQR)	57.5 (27.5–105.0)	49.0 (20.0–102.0)	.15
Male sex	273 (56.4)	133 (56.1)^a^	.95
HIV exposed	152 (31.4)	82 (34.5)	.41
HIV unexposed	330 (68.2)	151 (63.5)	.20
HIV exposure unknown	2 (0.4)	5 (2.1)	.04
	March 2015 to October 2015^b^		
	Induced Sputum and NPS Tested by PCR (n = 304)	No Induced Sputum and NPS Tested by PCR (n = 109)	
Age, d, at admission, median (IQR)	60.5 (42.0–94.0)	65.0 (41.0–111.0)	.27
Male sex	182 (60.5)^c^	63 (57.8)	.63
HIV exposed	106 (34.9)	34 (21.2)	.49
HIV unexposed	195 (64.1)	75 (68.8)	.38
HIV exposure unknown	3 (1.0)	0	.57

Data are presented as No. (%) unless otherwise indicated.

Abbreviations: HIV, human immunodeficiency virus; IQR, interquartile range; NPA, nasopharyngeal aspirate; NPS, nasopharyngeal swab.

^a^ Sex unknown for 1 infant.

^b^ Only infants 30–180 days old and admitted to medical wards are included.

^c^ Sex unknown for 3 infants.

Of the 484 infants who had both an NPS and NPA tested, 15 (3.1%) PCR-confirmed pertussis cases were identified; 13 (86.7%) were PCR positive on both samples, while 1 each was positive only on NPS or NPA (κ = 0.93; Table 2). The patient positive only on NPA had an “intermediate” result on NPS with Ct value for IS*481* of 37 and showing no amplification for *ptxS1*. The mean Ct value for IS*481* was similar between NPSs and NPAs on the 13 infants with a PCR-positive result on both samples (19.2 vs 21.3, respectively; *P* = .34).

**Table 2.** *Bordetella pertussis* Polymerase Chain Reaction Detection Using Different Respiratory Specimens

Parameter	NPS	NPA	Induced Sputum
Specimens tested, No.	711	484	304
Pertussis PCR-positive cases	19 (2.7%)	14 (2.9%)	10 (3.3%)
Pertussis PCR-positive cases in NPS and NPA^a^	13 + 1/15	13 + 1/15	…
Pertussis PCR-positive cases in NPS and induced sputum^a^	8 + 1/11	…	8 + 2/11
Sensitivity (95% CI)	86.4% (65.1%–97.1%)	93.3% (68.1%–99.8%)	83.3% (51.6%–97.9%)
Negative predictive value (95% CI)	99.6% (98.7%–99.9%)	99.8% (98.8%–100%)	99.3% (97.6%–99.9%)

Data are presented as No. unless otherwise indicated.

Abbreviations: CI, confidence interval; NPA, nasopharyngeal aspirate; NPS, nasopharyngeal swab; PCR, polymerase chain reaction.

^a^ No. of infants with positive PCR result on both samples + positive PCR result exclusively in the sample type described on the top row/combined positive PCR results from both samples.

From March to October 2015, 413 enrolled infants were eligible to have had induced sputum collected (ie, were ≥30 days old and were admitted to the general pediatric medical wards). Of these, 304 (73.6%) infants were tested with paired NPSs and induced sputum for *B. pertussis*. Among the infants eligible for induced sputum collection, no differences were detected between those in whom induced sputum was and was not collected (Table 1). Overall, PCR-confirmed pertussis was identified among 11 of the 304 (3.6%) infants who had both an NPS and induced sputum undertaken. This included 8 (72.7%) who were positive on both samples, 1 (9.1%) only positive on NPS, and 2 cases (18.2%) only positive on induced sputum (κ = 0.84; Table 2). One patient who was PCR positive only on induced sputum had an “intermediate” result on NPS with a Ct value for IS*481* of 35 and showing no amplification for *ptxS1*. The mean Ct value for IS*481* was similar between NPSs and induced sputum on the 8 infants who were PCR positive on both samples (23.3 vs 26.9, respectively; *P* = .24). Furthermore, of investigated cases in whom both an NPS and induced sputum were undertaken and among whom an NPA was also undertaken (n = 77), 1 additional case of pertussis was identified that was not identified by either NPS or induced sputum. Because the collection of induced sputum required the infants to be clinically stable, this meant that the collection of induced sputum occurred a mean of 1.4 days (standard deviation [SD], 2.0 days) after NPS collection. While NPS was collected on average 1.7 days (SD, 2.9 days) after admission, induced sputum was collected 3.1 days (SD, 3.4 days) after admission (*P* < .001).

PCR detection rates were similar using either NPSs (2.7%), NPAs (2.9%), or induced sputum (3.3%). The inclusion of positivity from any of the 3 sampling sites increased the identification of *B. pertussis* to 22 cases (3.1%). On considering these 22 infants as true pertussis PCR positives, the 3 types of specimens tested by PCR had similar NPV (>99%); sensitivity was also similar for NPSs (86.4%) compared with NPAs (93.3%; *P* = .63) and to induced sputum (83.3%; *P* = .99) (Table 2).

### *Bordetella pertussis* Culture Isolates

Detection of *B. pertussis* by culture was attempted in 274 induced sputum specimens, and 6 (2.2%) isolates were recovered compared to 10 (3.6%; *P* = .31) PCR-confirmed pertussis cases among the same infants. The sensitivity of culture on induced sputum was 60.0% (95% CI, 26.2%–87.8%) compared with PCR positivity. From infants <3 months of age, 148 NPSs were collected in Regan-Lowe media for bacterial culture, 2 (1.4%) of which yielded growth of *B. pertussis*. From this group of infants, 3 (n = 5 [3.4%]; *P* = .45) additional pertussis cases were detected by PCR. The sensitivity of culture using NPSs in Regan-Lowe was 40.0% (95% CI, 5.3%–85.3%) compared with PCR. Comparing the culture yield of *B. pertussis* between NPSs and induced sputum, of those infants who had both samples submitted for culture (n = 140), 3 (2.1%) were positive, of which 1 was identified on both samples and 1 each was positive only on NPS or induced sputum. All infants who were culture positive for pertussis were also pertussis PCR positive. Comparing the Ct values for IS*481* on the samples submitted for PCR from the infants who had cultures performed, the mean Ct value was lower on the 7 culture-positive infants compared with the 4 infants who were PCR positive but culture negative (21.0 vs 31.5, respectively; *P* = .015). Considering the clinical presentation of the 11 pertussis PCR-positive infants who had cultures performed, all presented with cough, but only 2 (1 culture positive and 1 culture negative) had a cough for >14 days, 3 had a paroxysmal cough with whoop (1 culture positive and 2 culture negative), 2 presented with posttussive vomit (1 culture positive and 1 culture negative), and a culture-negative infant presented with apnea.

## DISCUSSION

Accurate and timely diagnosis of *B. pertussis* infection is necessary to expedite appropriate treatment and improve patient care. In our study, respiratory specimens were collected over an 11-month period from hospitalized infants <6 months old and were tested for *B. pertussis* infection by an in-house real-time PCR method. The overall *B. pertussis–*positive rate was 3.1% and this was similar using NPS, NPA, or induced sputum as the tested specimen. Aspirates are normally the sample of choice for the detection of respiratory pathogens including *B. pertussis*; however, NPA collection is uncomfortable and laborious and requires skilled personnel [22]. During the initial period of the study we sought to compare the utility of NPAs over NPSs for the detection of *B. pertussis*. From November 2014 to May 2015, paired NPAs and NPSs were tested; after we found that nasopharyngeal samples collected with flocked swabs were an excellent alternative to NPAs based on being less invasive and with comparable sensitivity, NPA collection was stopped.

Although it has been suggested that specimens that lack the collection of ciliated epithelium cells such as throat washes, anterior nose swabs, and sputum are not adequate for *B. pertussis* testing [23, 24], the utility of induced sputum for the detection of pertussis has recently been reported in South Africa [19, 20]. In our study, however, no significant added value of induced sputum testing compared to NPSs was noticed; this difference may result from the different PCR protocols used and from the fact that the infants recruited to our study were in general younger. In a case-control study of children with pneumonia, with cases having a median age of 5 months, *B. pertussis* detection was performed on NPSs and induced sputum by PCR using a commercial kit (FTDResp33) that detects the presence of IS*481* [20]. In this study, 75% of all pertussis cases were detected by induced sputum only; however, as described below, the use of a single PCR target is not ideal for *B. pertussis* testing. In another report from South Africa, children hospitalized with LRTI were also tested for *B. pertussis* on NPS and induced sputum using a dual-target commercial kit (LightMix), and induced sputum testing increased the detection yield of *B. pertussis* over NPS alone by 50% [19]. The study participants were, however, <13 years old, with a median age of 8 months, and only 9% were <2 months of age compared with 50% in our study. Another fact that might contribute to the difference observed in the induced sputum results is that in our study, induced sputum was only collected when infants were clinically stable, at a mean of 1.4 days after NPS collection, and as reported in the study by Muloiwa et al [19], participants positive for *B. pertussis* on induced sputum only had a shorter duration of symptoms compared with those who were *B. pertussis* positive on NPS, suggesting that testing of induced sputum is more likely to be positive very early in the course of the infection.

*Bordetella pertussis* culture has been considered the gold standard as it is 100% specific; however, its sensitivity, as also observed by us, is poor to modest, ranging from 12% to 60% [6, 25, 26]. Although our numbers were small, the Ct values for IS*481* detected in respiratory samples tested by PCR were lower in samples from which *B. pertussis* isolates were recovered by culture compared with samples where culture was attempted but no *B. pertussis* was recovered, suggesting that higher bacterial load is required for culture isolation. In our study, a pertussis case was defined as an enrolled infant with a positive PCR or culture result from a respiratory specimen independent of clinical presentation. Comparing the classical symptoms of pertussis (ie, prolonged cough, paroxysmal cough, posttussive vomiting) between infants who tested pertussis PCR and culture positive and infants who were pertussis PCR positive but culture negative, no differences were noted, although the number of infants with classical pertussis symptoms was low. Due to the low sensitivity of culture, considering this method as the gold standard to evaluate new assays is not desirable. In our analysis we used a composite reference standard to compare the sensitivity of the different specimens, and comparable sensitivities by PCR testing were detected for the 3 different types of respiratory samples.

Besides the appropriate clinical specimen, accurate PCR testing is also dependent on the primer selection, amplification conditions, and internal and external positive and negative controls [9, 27]. In our study, samples were initially screened for the presence of the IS*481* target, and if this insertion sequence was detected, the presence of pertussis toxin was investigated. Because IS*481* target is present in multiple copies in the *B. pertussis* genome, this PCR is highly sensitive [28]; however, because IS*481* is also present in lower copy numbers in the *Bordetella holmesii* genome and in some human and animal *Bordetella bronchiseptica* isolates, a stand-alone IS*481* PCR has limitations for detection of *B. pertussis* [29, 30]. The dual-target PCR, the evaluation of the Ct values of both targets to attribute positivity, and the careful inclusion of positive and negative controls allowed us to identify *B. pertussis* with high specificity [9].

Limitations of our study include that not all patients had the 3 types of specimens tested by PCR, limiting the comparisons to 2 time periods. Also due to the laborious culture procedure, culture was only undertaken in less than half of the participants. We restricted the collection of induced sputum to infants aged >1 month because it is a difficult specimen to obtain from neonates. Nevertheless, because the highest burden of pertussis disease is in very young infants, and induced sputum cannot be collected, there is probably a limited role for induced sputum in the diagnosis of pertussis in this age group.

In conclusion, in our study NPS, NPA, and induced sputum performed similarly for the detection of *B. pertussis* infection in young infants by PCR, suggesting that to improve pertussis testing, the type of sample collected is probably less important than standardization of the PCR assay that may eventually replace bacterial culture as the gold standard.

## Supplementary Data

Supplementary materials are available at http://cid.oxfordjournals.org. Consisting of data provided by the author to benefit the reader, the posted materials are not copyedited and are the sole responsibility of the author, so questions or comments should be addressed to the author.

Supplementary Data
