# Supplementary material for: Comparing the Yield of Nasopharyngeal Swabs, Nasal Aspirates, and Induced Sputum for Detection of Bordetella pertussis in Hospitalized Infants
Source: Clin Infect Dis. 2016 Nov 2;63(Suppl 4):S181–6. doi: 10.1093/cid/ciw521 (PMC5106614; doi:10.1093/cid/ciw521)
Supplement: Supplementary Data [file supp_63_suppl-4_S181__index.html]

Supplementary Data 

# Comparing the Yield of Nasopharyngeal Swabs, Nasal Aspirates, and Induced Sputum for Detection of *Bordetella pertussis* in Hospitalized Infants

## Supplementary Data

Supplementary Data

- Supplementary Data - Docx file
