# Supplementary material for: Comparing the Yield of Nasopharyngeal Swabs, Nasal Aspirates, and Induced Sputum for Detection of Bordetella pertussis in Hospitalized Infants
Source: Clin Infect Dis. 2016 Nov 2;63(Suppl 4):S181–6. doi: 10.1093/cid/ciw521 (PMC5106614; doi:10.1093/cid/ciw521)
Supplement: Supplementary Data [file supp_ciw521_ciw521supp.docx]

**Supplementary Table 1. Primers and Probes sequences**

| Gene Name | Sequence (5’-3’) |
| --- | --- |
| *IS481* | For 5’-CAAGGCCGAACGCTTCAT-3’  Rev 5’-GAGTTCTGGTAGGTGTGAGCGTAA-3’  Probe 5’-NED-CAGTCGGCCTTGCGTGAGTGGG-MGB-3’ |
| *PtxS* | For 5’-CGCCAGCTCGTACTTC-3’  Rev 5’-GATACGGCCGGCATT-3’  Probe 5’-VIC-AATACGTCGACACTTATGGCGA-MGB-3’ |
| *hIS1001* | For 5’- GGCGACAGCGAGACAGAATC -3’ |
|  | Rev 5’- GCCGCCTTGGCTCACTT -3’ |
|  | Probe 5’-VIC- CGTGCAGATAGGCTTTTAGCTTGAGCGC–MGB-3’ |
| *pIS1001* | For 5’- TCGAACGCGTGGAATGG -3’ |
|  | Rev 5’- GGCCGTTGGCTTCAAATAGA -3’ |
|  | Probe 5’-FAM- AGACCCAGGGCGCACGCTGTC-MGB3’ |
|  | For 5’-AGATTTGGACCTGCGAGCG-3’ |
| Human *rnaseP* | Rev 5’-GAGCGGCTGTCTCCACAAGT-3’ |
|  | Probe 5’-FAM-TTCTGACCTGAAGGCTCTGCGCG-MGB3’ |
|  | For 5’- GCTCCTCCTGTTCGACAGTCA -3’ |
| Human *GAPDH* | Rev 5’- ACCTTCCCCATGGTGTCTGA -3’ |
|  | Probe 5’-NED-CGTCGCCAGCCGAGCCACA -MGB3’ |

**Supplementary Table 2. PCR results interpretation**

| Targets | | | | Interpretation |
| --- | --- | --- | --- | --- |
| *IS481* | p*IS1001* | h*IS1001* | *ptx* S1 |  |
| CT<35 | - | - | + or - | *B*. *pertussis* |
| CT ≤35-<40 | - | - | + | *B*. *pertussis* |
| CT ≤35-<40 | - | - | - | indeterminate |
| Positive | - | + | - | *B*. *holmesii* |
